# Exercise Training and Grape Seed Extract Co-Administration Improves Lipid Profile, Weight Loss, Bradycardia, and Hypotension of STZ-Induced Diabetic Rats

**Published:** 2013-12-01

**Authors:** Mohammad Badavi, Hassan Ali Abedi, Mahin Dianat, Ali Reza Sarkaki

**Affiliations:** 1 Physiology Research Center, Physiology Department, Faculty of Medicine, Ahwaz Jundishapour University of Medical Sciences, Ahwaz, IR Iran; 2 Physiology Department, Faculty of Medicine, Jahrom University of Medical Sciences, Jahrom, IR Iran

**Keywords:** Exercise, Grape Seed Extract, Streptozotocin, Bradycardia, Hypotension

## Abstract

**Background:**

Exercise Training (ET) and Grape Seed Extract (GSE) as an antioxidant have many positive effects on controlling diabetes mellitus and its complications.

**Objectives:**

This study aimed to determine the effects of GSE alone or combined with ET on body weight, plasma lipid profile, blood pressure, and heart rate in STZ-induced diabetic rats.

**Methods:**

In this study, male Wistar rats were randomly assigned to five groups: sedentary control, sedentary diabetic, trained diabetic, GSE treated sedentary diabetic, and GSE treated trained diabetic. ET was conducted on the treadmill daily for 8 weeks. One way ANOVA followed by LSD test was used for statistical analysis.

**Results:**

Reduction of body weight, high density lipoproteins, heart rate, and systolic blood pressure and increment of total cholesterol, triglyceride, low density lipoprotein, and very low density lipoproteins were observed after STZ injection. Co-administration of GSE and ET had more positive effects on lipid profile compared to each method alone. In addition, GSE and ET modified heart rate partially, while their combination was more effective in improvement of heart rat in conscious rats. On the other hand, administration of ET or GSE alone did not affect systolic blood pressure and body weight, while their combination restored systolic blood pressure completely and improved body weight partially.

**Conclusions:**

The study findings indicated that ET combined with GSE had more beneficial effects compared to each one alone on the complications of STZ induced diabetes. This may constitute a convenient and inexpensive therapeutic approach to diabetic complications.

## 1. Background

Injection of Streptozotocin (STZ), the most commonly used agents in experimental diabetes (1), to rats produces a diabetic state characterized by hyperglycemia, loss of weight, hypotension, bradycardia (2, 3), increase in plasma Total Cholesterol (TC), Triglycerides (TG), Low-Density Lipoprotein cholesterol (LDL-c), Very Low-Density Lipoprotein cholesterol (VLDL-c), and decrease in High Density Lipoprotein (HDL) (4).

In management of diabetes and its complications, Exercise Training (ET) has many positive effects, such as increase in insulin sensitivity, decrease in glycosylated hemoglobin (HbA1c), improvement of blood lipid profiles and blood pressure (5), and improvement of systemic vascular resistance and heart rate (6). It has also been shown that the incidence of cardiovascular morbidity and mortality during diabetes was reduced by ET (7). In addition, ten weeks ET reversed hypotension and bradycardia induced by STZ in rats (8).

Since oxidative stress contributes to complications of Diabetes Mellitus (DM) (9-12), pharmacological agents that ameliorate oxidative stress may improve diabetes and its complications. It has been reported that treatment of diabetic hypertensive rats with vitamin E decreased blood pressure (13).

Grape Seed Extract (GSE) has many favorable effects on human health, such as lowering of LDL-c, reduction of Cardiovascular Diseases (CVD), and scavenging of free radicals (14). The antioxidant power of proanthocyanidins of the grape seeds is twenty times greater than vitamin E and fifty times greater than vitamin C (14). Badavi et al. showed that GSE improved hypertension and heart rate induced by lead exposure (15).

However, to our knowledge, no studies have been conducted on the effects of GSE alone or combined with exercise on the lipid profile, body weight, heart rate, and blood pressure of diabetic models. Therefore, the current study aims to determine the effects of GSE alone or combined with ET on the lipid profile, body weight, heart rate, and blood pressure of STZ-induced diabetic rats.

## 2. Materials and Methods

### 2.1. Animals and Treatment

In this study, 45 male Wistar rats weighing 200 - 240 g were obtained from the animal house of physiology research center at Ahwaz Jundishapour University of Medical Sciences, Ahwaz, Iran. The animals were randomly assigned to five groups each containing 9 rats: Sedentary Control (SC), Sedentary Diabetic (SD), Trained Diabetic (TrD), sedentary diabetic treated with GSE (ExD), and trained diabetic treated with GSE (TrExD). GSE was dissolved in 1 mL distilled water and administered orally via gavage needle once a day. The duration of the protocol was 8 weeks. Diabetes was induced by a single intraperitoneal injection of STZ (60 mg / kg body weight) dissolved in 0.3 mL normal saline (16). The experimental protocol and procedures were submitted and approved by the Institutional Animal Care and Use Committee of the University.

### 2.2. Drugs

STZ was obtained from Sigma (St. Louis, Mo). Besides, Ketamine and Xylazine were prepared by Alfasan Co (Woderen-Holland).

### 2.3. Exercise Training Protocol

The rats performed ET on treadmill daily for 8 weeks, 1 day after the diabetic state was verified as shown in Table 1. 

**Table 1.** Exercise Training Protocol for the Rats on the Treadmill

Week	Belt Speed (m / min)	Inclination (˚)	Total Time (min)
**1**	16	0	30
**2**	16	5	30
**3**	16	10	45
**4**	16	12	45
**5**	16	12	60
**6**	16	12	60
**7**	16	12	60
**8**	16	12	60

### 2.4. Preparation of Grape Seed Extract

VitisVinifera grape seeds were confirmed by Qazvin Agricultural Research Center, Qazvin, Iran. Voucher specimen was available in the herbariumat the Department of Phamacognosy, Faculty of Pharmacy, Ahvaz Jundishapur University of Medical Sciences, Ahwaz, Iran. Grape seeds were separated from the grapes manually, air-dried (in the shade, 25 - 30°C) for one week, and milled to fine powder. The grape seed powder was macerated in 70% ethanol (25% w/v) for three days at room temperature and was stirred three times a day. Then, the mixture was filtered with cheese cloth, the filtrate was dried at room temperature (about 25°C) to evaporate ethanol, and GSE was obtained (25 - 30 %) as a powder (17).

### 2.5. Plasma Lipid Profile Determination

Immediately after cardiac puncture under anesthesia with ketamine and xylasine, blood samples were obtained from the heart and transferred into EDTA containing tubes. The samples were then centrifuged at 4000 g for 10 min to obtain plasma. Afterwards, the plasma was stored at -70°C for biochemical analysis. Enzymatic colorimetric methods (Pars Azmune, Tehran, Iran) were used for measurement of TC and TG levels. In addition, HDL-c was determined by enzymatic colorimetric method (Pars Azmune) after precipitation of non- HDL-c lipoproteins by phosphotungstic acid and magnesium chloride in the plasma. Besides, VLDL was calculated as follows (18):

VLDL = Total serum triglycerides / 5

Moreover, LDL-c was calculated based on Friedwald’s equation (19) for less than 400 mg / dL TG-containing samples:

LDL-c = Total serum cholesterol–(VLDL–Total serum HDL).

### 2.6. Heart Rate and Blood Pressure Recording

Heart rate and blood pressure were recorded once a week. Prior to blood pressure and heart rate measurement, conscious rats were placed in a restrainer, pre-warmed, and allowed to rest for about 25 min. Then, these variables were determined by tail plethysmography coupled to a computer system (Powerlab, AD Instrument, Australia). Overall, three consecutive recordings (at least 5 min apart) were performed and the average of the recordings was calculated for each rat (15).

### 2.7. Statistical Analysis

The results were expressed as mean ± SEM. The data were first analyzed for normal distribution using kolmogrov-smirnof test. Then, comparisons were made between the study groups using one way and repeated measures ANOVA followed by LSD tests. Besides, P values < 0.05 were considered as statistically significant.

## 3. Results

### 3.1. Blood Lipid Profile in Different Groups

Eight weeks after STZ injection, significant changes were observed in the plasma lipid profile, such as lowered HDL-c, and elevated TC, TG, LDL-c, and VLDL (P < 0.001) (Table 2). However, ET improved HDL-c (P = 0.006), TC, TG, LDL-c, and VLDL (P < 0.001). In addition, GSE had improving effects on TC (P < 0.001), HDL-c (P = 0.001), and LDL-c (P = 0.001), but did not change TG and VLDL. On the other hand, in comparison to ET or GSE alone, co-administration of GSE and ET had more improving effects on TC, TG, HDL-c, LDL-c, and VLDL (P < 0.001). Moreover, plasma lipid profile values in the GSE + ET treated diabetic animals were the same as the corresponding values of the sedentary control group. 

**Table 2.** Plasma Lipid Profile in Different Groups after 8 Weeks (Mean ± SEM, n = 6 - 7)[Table-fn fn6695]

Parameter Groups	TC (mg / dL)	TG (mg / dL)	HDLc (mg / dL)	LDLc (mg / dL)	VLDL (mg / dL)
**SC**	79.33 ± 3.77	64.5 ± 5.55	47.83 ± 1.97	18.6 ± 1.97	12.9 ± 1.11
**SD**	163.86 ± 7.18 ^a^	157.14 ± 7.68 ^a^	33.29 ± 1.51 ^a^	99.14 ± 5.45 ^a^	31.43 ± 1.54 ^a^
**TrD**	112 ± 6.93 ^a, b^	117.5 ± 7.74 ^a, b^	41.5 ± 2.26 ^a, b^	47 ± 4.83 ^a, b^	23.5 ± 1.55 ^a, b^
**ExD**	134.43 ± 6.86 ^a, b^	140.29 ± 5.98 ^a^	43.14 ± 1.77 ^b^	63.23 ± 6.49 ^a, b^	28.057 ± 1.2 ^a^
**TrExD**	87.43 ± 6.72 ^b^	79.71 ± 4.47 ^b^	51.57 ± 2.21 ^b^	19.91 ± 7.28 ^b^	15.94 ± 0.89 ^b^

Abbreviations: TC, total cholesterol; TG, triglycerides; HDLc, high-density lipoprotein cholesterol; LDLc, low-density lipoprotein cholesterol; VLDL, very low-density lipoprotein cholesterol; SC, sedentary control; SD, sedentary diabetic;TrD, trained diabetic;ExD, sedentary diabetic that received grape seed extract; TrExD, trained diabetic that received grape seed extract

^a^P < 0.05 significantly different from SC,

^b^P < 0.05 significantly different from SD (One-Way ANOVA followed by LSD multiple comparison tests)

### 3.2. Body Weight Changes

**Figure 1.**
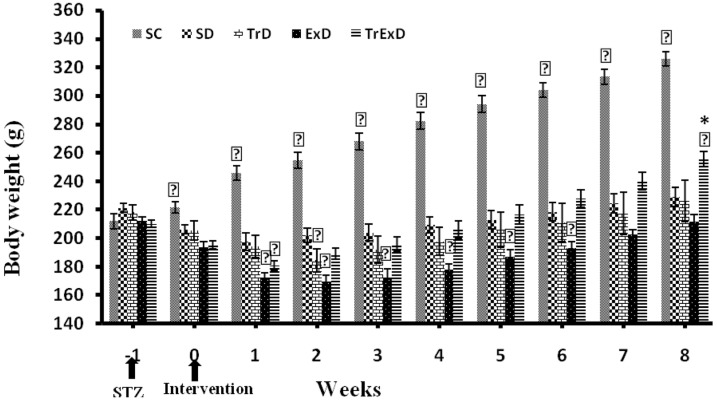
Weekly Body Weight Changes (Mean Â ± SEM, n = 7 - 8) in Different Groups During 8 Weeks Abbreviations: SC, sedentary control; SD, sedentary diabetic; TrD, trained diabetic; ExD, GSE treated sedentary diabetic; TrExD, GSE treated trained diabetic *P < 0.05 significantly different from SC, † P < 0.05 significantly different from SD (repeated measurement ANOVA followed by LSD multiple comparison tests)

At the beginning of the experiment, no significant difference was found among the study groups regarding their body weight (Figure 1). However, the control groups gained weight throughout 9 weeks. On the other hand, the body weight of the sedentary diabetic group reduced during the first 2 weeks after STZ injection. In trained diabetic and GSE treated sedentary diabetic groups also, this reduction continued for 3 weeks and then increased slightly to reach the pre-STZ values at week 8. Nevertheless, the body weight of the GSE + ET treated diabetic group reduced initially during the first 2 weeks after STZ injection, then began to increase throughout the next 7 weeks, and reached a value that was significantly different from the pre-STZ value (P < 0.001) as well as from the control group (Figure 1). 

### 3.3. Heart Rate and Systolic Blood Pressure

**Figure 2.**
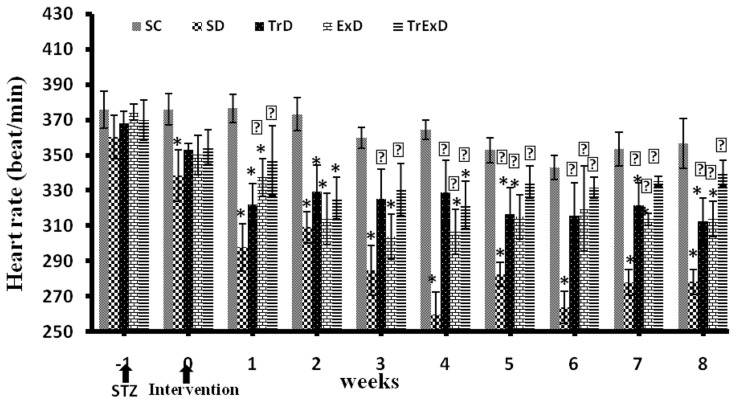
Weekly Heart Rate Changes (Mean Â ± SEM, n = 7 - 8) in Different Groups During 8 Weeks Abbreviations: SC, sedentary control, SD, sedentary diabetic, TrD, trained diabetic, ExD, mGSE treated sedentary diabetic, TrExD, GSE treated trained diabetic *P < 0.05 significantly different from SC, †P < 0.05 significantly different from SD (repeated measurement ANOVA followed by LSD multiple comparison tests)

As shown in Figure 2, STZ-induced diabetes decreased heart rate in all the groups during the first 2 – 3 weeks post injection. However, the observed bradycardia was more severe in the sedentary diabetic group compared to the trained diabetic and GSE treated sedentary diabetic groups. At the end of the protocol, the heart rate in the sedentary diabetic group was significantly lower than that of the sedentary control group. Therefore, administration of ET or GSE partially and combination of ET and GSE completely improved the heart rate. However, no significant change was found in the control heart rate throughout the experiment. 

**Figure 3.**
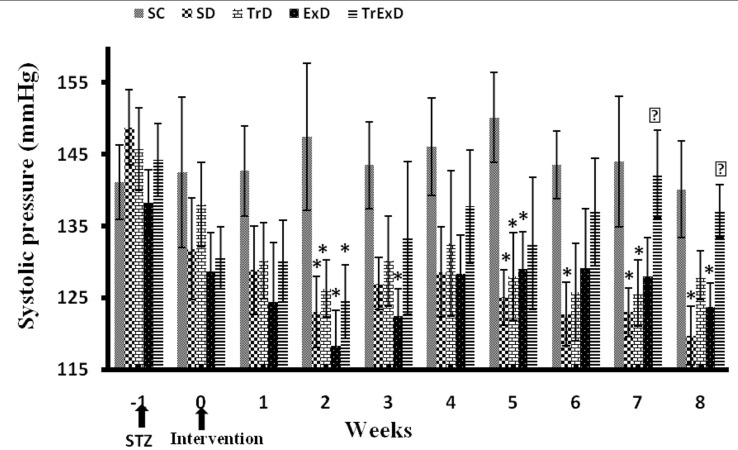
Weekly Systolic Blood Pressure Changes (Mean Â ± SEM, n = 7 - 8) in Different Groups During 8 Weeks Abbreviations: SC, sedentary control, SD, sedentary diabetic, TrD, trained diabetic, ExD, GSE treated sedentary diabetic, TrExD, GSE treated trained diabetic *P < 0.05 significantly different from SC, †P < 0.05 significantly different from SD (repeated measurement ANOVA followed by LSD multiple comparison tests)

In comparison to the control group, the Systolic Blood Pressure (SBP) decreased in all the groups that received STZ in the first 2 weeks after diabetes induction (Figure 3). Although ET or GSE administration alone did not restore SBP, the combination of ET and GSE could restore it toward the control group. 

## 4. Discussion

As expected, body weight, HDL-c, heart rate, and SBP reduced and plasma TC, TG, LDL-c, and VLDL increased significantly in diabetic animals induced by STZ, which is in accordance with other studies conducted on the issue (2-4). Administration of ET or GSE alone improved heart rate partially, while the combination of GSE and ET improved them completely in conscious rats. On the other hand, administration of ET or GSE alone did not affect SBP and body weight, while their combination restored SBP completely and improved body weight partially. In general, control of blood lipid level is an important step towards decreasing the incidence of long-term complications of DM (19). In this study, combined ET and GSE were more effective than ET or GSE alone in improvement of lipid profiles in diabetic state induced by STZ.

In line with our study results, previous studies showed the positive effects of exercise on thelipid profiles of healthy rats (20,21) as well as type 2 (22) and type 1 (23) diabetic rats. Positive changes in lipid profile induced by ET in the trained diabetic animals may occur via an increase in triglyceride lipolysis (24), improvement of antioxidant/oxidant ratio (25), and altered synthesis of LDL-C or removal rate of LDL-C from the plasma by the tissues (20). In general, antioxidants play a major role in prevention of diabetes and its complications by free radicals scavenging (26). It has been shown that vitamin C (27), vitamin E (28), and α lipoic acid (19) could improve blood lipid profile in diabetic rats. Moreover, vitamin C was reported to prevent LDL-cholesterol from oxidative damage by scavenging the free radicals, helping the degradation of cholesterol, and directing the cholesterol towards bile acid synthesis (27). In the present study, GSE as an antioxidant significantly improved lipid profile in diabetic animals. One study also indicated that grape seed tannins enhanced reverse cholesterol transport and bile acid excretion and reduced intestinal cholesterol absorption in the rats fed by hypercholesterolemic diets (29).

The combination of these two factors could potentiate these favorable effects, but the exact mechanisms and the involved systems are needed to be determined.

Loss of weight induced by STZ could be related to the inability to metabolize the carbohydrates that shift fuel sources to fatty acids (3) and proteins (30) as an energy source. Therefore, wasting of proteins and fatty acid stores induced by insulin deficiency might lead to reduction of body weight. It seems that co-administration of ET and GSE increased body weight by improvement of carbohydrate metabolization although more studies are required to be conducted on the issue.

The mechanisms related to STZ induced bradycardia in diabetic rats may include dysfunction of intrinsic and/or extrinsic control mechanisms to the heart (3). Reduction in the heart rate following STZ injection in isolated perfused hearts shows defect in intrinsic mechanisms, while that in conscious rats shows defect in intrinsic and/or extrinsic mechanisms that control the heart rate (3). Studies on isolated cardiac preparation indicated that basal spontaneous pacemaker rate was reduced by STZ. Thus, the reduced rate may reflect changes in electrophysiological properties of the sino-atrial node, such as alteration in maximum diastolic potential or the threshold or slope of diastolic depolarization (31). The observed bradycardia could also be mediated in part by alteration in autonomic nervous system; an increase in vagal or a decline in sympathetic tone would diminish heart rate. Previous results suggested that both sympathetic and parasympathetic tone to the heart were reduced significantly in diabetic animals (31, 32). The individuals with parasympathetic dysfunction have a high resting heart rate most likely because of vagal neuropathy that results in unopposed increased sympathetic tone. Moreover, combined parasympathetic - sympathetic dysfunction causes slower heart rates. Yet, advanced nerve dysfunction fixes heart rate (33). Additionally, increased expression of inducible Nitric Oxide Synthase (iNOS) and oxidative stress by chronic diabetes may produce peroxynitrite/nitrotyrosine and cause nitrosative stress leading to cardiovascular depression, bradycardia, and hypotension in STZ-induced diabetic rats (34).

The decrease in blood pressure may be explained by a study by Jackson and Carrier (35). They suggested that the reduction in arterial pressure may be the result of a decreased cardiac output in diabetic sedentary rats due to hypovolemia caused by hyperglycemicosmotic diuresis. Furthermore, increased parasympathetic nervous system activity can cause hypotension in the diabetic group although De Angelis (36) demonstrated a decrease in vagal function suggesting that changes in arterial pressure are not related to an increase in parasympathetic activity. The hemodynamic effect could be associated with the rapid and steady increase in plasma concentrations of nitric oxide, one of the most potent endothelium- derived relaxing factors (37,38). Although exercise or grape seed could not significantly increase arterial pressure, the combination of exercise and grape seed extract attenuated the STZ- induced hypotension and improved it toward the values observed in the controls. These protective effects may be attributable to the improvement of nitrosative stress (34), amelioration of diabetic-induced oxidant/antioxidant levels (39), changes in peripheral resistance (40), a better ventricular contractility, enhanced resting HR (8, 36), improvement of glucose homeostasis (36), arterial compliance improvement (41), and conduit arterial elasticity (42).

In conclusion, the study findings indicated that STZ-induced diabetes significantly reduced body weight, high density lipoproteins, heart rate, and SBP and increased total cholesterol, triglyceride, low density lipoprotein, and very low density lipoprotein. Moreover, grape seed extract combined with exercise training had a more significant improving effect on theplasma lipid profile, body weight, heart rate, and SBP compared to exercise training or grape seed extract alone. Thus, it may constitute a convenient and inexpensive therapeutic approach to some diabetic complications.
